# Statistical assessment of nonpoint source pollution in agricultural watersheds in the Lower Grand River watershed, MO, USA

**DOI:** 10.1007/s11356-018-3682-7

**Published:** 2018-11-14

**Authors:** Fadhil K. Jabbar, Katherine Grote

**Affiliations:** 1 Department of Geosciences and Geological and Petroleum Engineering, Missouri University of Science and Technology, McNutt Hall, 1400 N. Bishop Ave, Rolla, MO 65401 USA; 2 College of Science, University of Misan, Amarah, Iraq

**Keywords:** Agricultural pollution, Nutrients, Surface water quality, Lower Grand River Watershed, Biotic index, Statistical analysis, PCA

## Abstract

The water quality in many Midwestern streams and lakes is negatively impacted by agricultural activities. Although the agricultural inputs that degrade water quality are well known, the impact of these inputs varies as a function of geologic and topographic parameters. To better understand how a range of land use, geologic, and topographic factors affect water quality in Midwestern watersheds, we sampled surface water quality parameters, including nitrate, phosphate, dissolved oxygen, turbidity, bacteria, pH, specific conductance, temperature, and biotic index (BI) in 35 independent sub-watersheds within the Lower Grand River Watershed in northern Missouri. For each sub-watershed, the land use/land cover, soil texture, depth to bedrock, depth to the water table, recent precipitation area, total stream length, watershed shape/relief ratio, topographic complexity, mean elevation, and slope were determined. Water quality sampling was conducted twice: in the spring and in the late summer/early fall. A pairwise comparison of water quality parameters acquired in the fall and spring showed that each of these factors varies considerably with season, suggesting that the timing is critical when comparing water quality indicators. Correlation analysis between water quality indicators and watershed characteristics revealed that both geologic and land use characteristics correlated significantly with water quality parameters. The water quality index had the highest correlation with the biotic index during the spring, implying that the lower water quality conditions observed in the spring might be more representative of the longer-term water quality conditions in these watersheds than the higher quality conditions observed in the fall. An assessment of macroinvertebrates indicated that the biotic index was primarily influenced by nutrient loading due to excessive amounts of phosphorus (P) and nitrogen (N) discharge from agricultural land uses. The PCA analysis found a correlation between turbidity, *E. coli*, and BI, suggesting that livestock grazing may adversely affect the water quality in this watershed. Moreover, this analysis found that N, P, and SC contribute greatly to the observed water quality variability. The results of this study can be used to improve decision-making strategies to improve water quality for the entire river basin.

## Introduction

Nonpoint source (NPS) pollution from agricultural activities has become the main source of contamination in surface water in the USA. In much of the US Midwest, agriculture was identified as the most likely source to cause impairment in the assessed rivers and streams (USEPA 2013). The primary pollutants from agricultural activities are excessive inputs of nutrients through commercial fertilizer and manure (Ahearn et al. 2005; Fournier et al. 2017; Chen et al. 2017; Kourgialas et al. 2017), runoff from pesticides and herbicides (Hildebrandt et al. 2008; Sangchan et al. 2013; Cruzeiro et al. 2015), and increased turbidity due to soil erosion (Zhang and Huang 2014). The most problematic nutrients are phosphorus (P) and nitrogen (N), which are often carried into streams through overland flow during rainfall events (Driscoll et al. 2003; Maillard and Santos 2008; Kato et al. 2009; Mouri et al. 2011; Yu et al. 2015), especially before the growing season and after harvest (Zhu et al. 2012). Excessive inputs of nutrients, such as nitrogen and phosphorus, to surface water can contribute to eutrophication, excessive algal growth, increased toxicity, and other adverse influences on fish and aquatic invertebrate communities (Xu et al. 2013; Wang and Tan 2017). Generally, all types of agricultural practices and land use, including animal feeding operations (AFOs), are treated as agricultural NPS pollution. NPS pollution depends on hydrological conditions and is difficult to measure or control directly. However, due to the features of NPS pollution, field measurements, and the limitations of experiments, NPS pollution management practices depend on spatial-temporal simulation modeling, a key method used to estimate NPS pollution related to spatial uncertainty (Shamshad et al. 2008; Huiliang et al. 2015). Various approaches have been used to estimate the loads of NPS pollution, including small spatial-scale experiments and watershed-scale modeling, which accurately calculates the pollution loads of different land uses through experimental methods (Alberti et al. 2007; Pratt and Chang 2012). Thus, the methods used in field experimental methods are too time-intensive and expensive to translate into practical applications (Liang et al. 2008). Furthermore, it is difficult to extend field experimental methods to the watershed scale due to the biological and chemical reactions and the complexity of the transport mechanism in the watershed.

Some research has tried to investigate the impacts of land use and land cover on surface water quality (Haidary et al. 2013; Huang et al. 2015). The relationship between land cover and water quality has been studied to reveal the effects of the characteristics of watersheds on the dissolved oxygen (DO) turbidity and river temperature (Li et al. 2015). Other research analyzed the watershed scale in addition to using remotely sensed data and GIS as well as multivariate analysis to estimate the influence of the land cover on the nutrients, suspended sediments, and ecological integrity of rivers (Lai et al. 2011; Exner-Kittridge et al. 2016). For example, when studying largely forested watersheds in North Carolina, Potter et al. (2005) applied simple regression and stepwise regression to develop relationships between eight independent variables (derived from land use/land cover (LULC) and landform characteristics) and the macroinvertebrate index. Schoonover and Lockaby (2006) and Rothenberger et al. (2009) used a similar method to develop correlations between LULC parameters (e.g., percent of impervious surface, mixed forest, evergreen forest, and pasture) and quality parameters (e.g., nutrient and bacteriological characteristics) for watersheds in the USA. Because a large number of variables are required to describe water quality and the factors that affect it, multivariate statistical analysis has become a powerful tool to investigate and interpret the results. Among the multivariate analysis approaches, principal component analysis (PCA) has been widely used to determine how different reaches of a stream contributes to the overall pollution load (Kannel et al. 2007; Bu et al. 2010; Olsen et al. 2012) or which parameters are most crucial in calculating the water quality index (WQI) (Sharma and Kansal 2011; Koçer and Sevgili 2014; Zeinalzadeh and Rezaei 2017). Furthermore, PCA analysis can also illustrate how the variability of water quality properties changes with time (Ouyang et al. 2006; Jung et al. 2016).

Therefore, this study builds upon the results of previous research by developing correlations in a large number (35) of independent watersheds with mixed LULC (including forest, pasture, row crops, and urban areas) and investigating which combinations of LULC, geologic, and topographic properties are most predictive of both the physicochemical water quality parameters and the biotic index. The independent variables in these relationships are readily available GIS-based parameters. Although similar or more accurate results can be obtained using surface water models, such as the Soil and Water Assessment Tool or BASINS, these models require more sophisticated or temporally variable inputs than the relationships developed in this study, and thus, are much more difficult to implement.

The primary objectives of this study are to provide relationships that can be used with readily available GIS databases and ArcGIS tools to indicate which watersheds have the combination of characteristics most likely to result in poor water quality, to assess regionally variability in water quality parameters both spatially and temporally, and to determine which water quality characteristics have the greatest impact on aquatic health. Scientists and regulators can use these results to inform sampling campaigns or to identify areas where more sophisticated modeling is appropriate.

## Methods and materials

### Site background

**Fig. 1:**
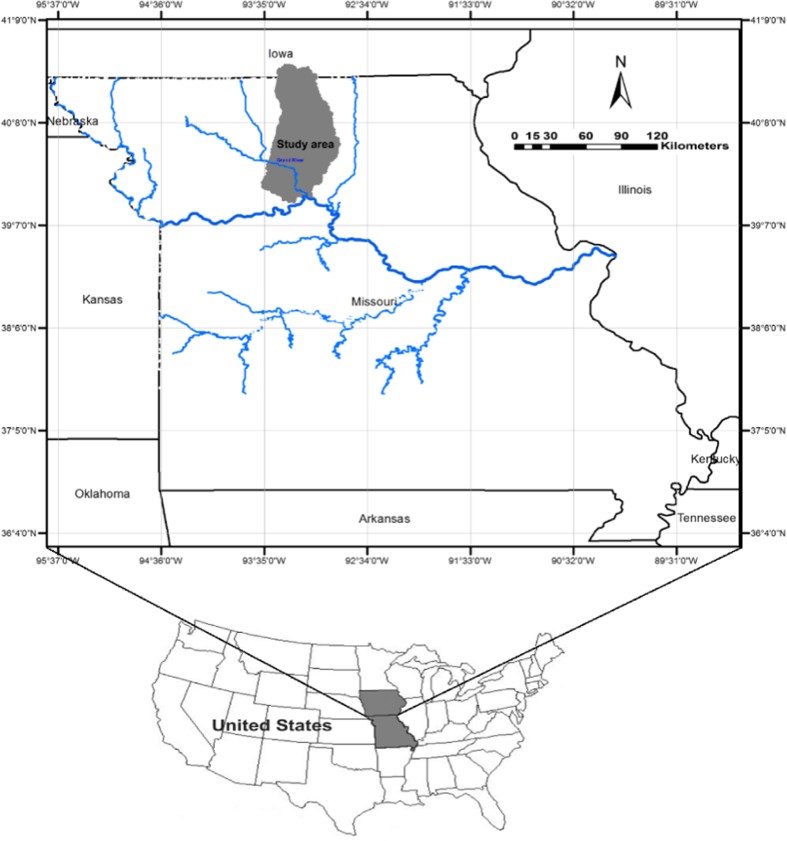
The location of the Lower Grand River Watershed

**Fig. 2:**
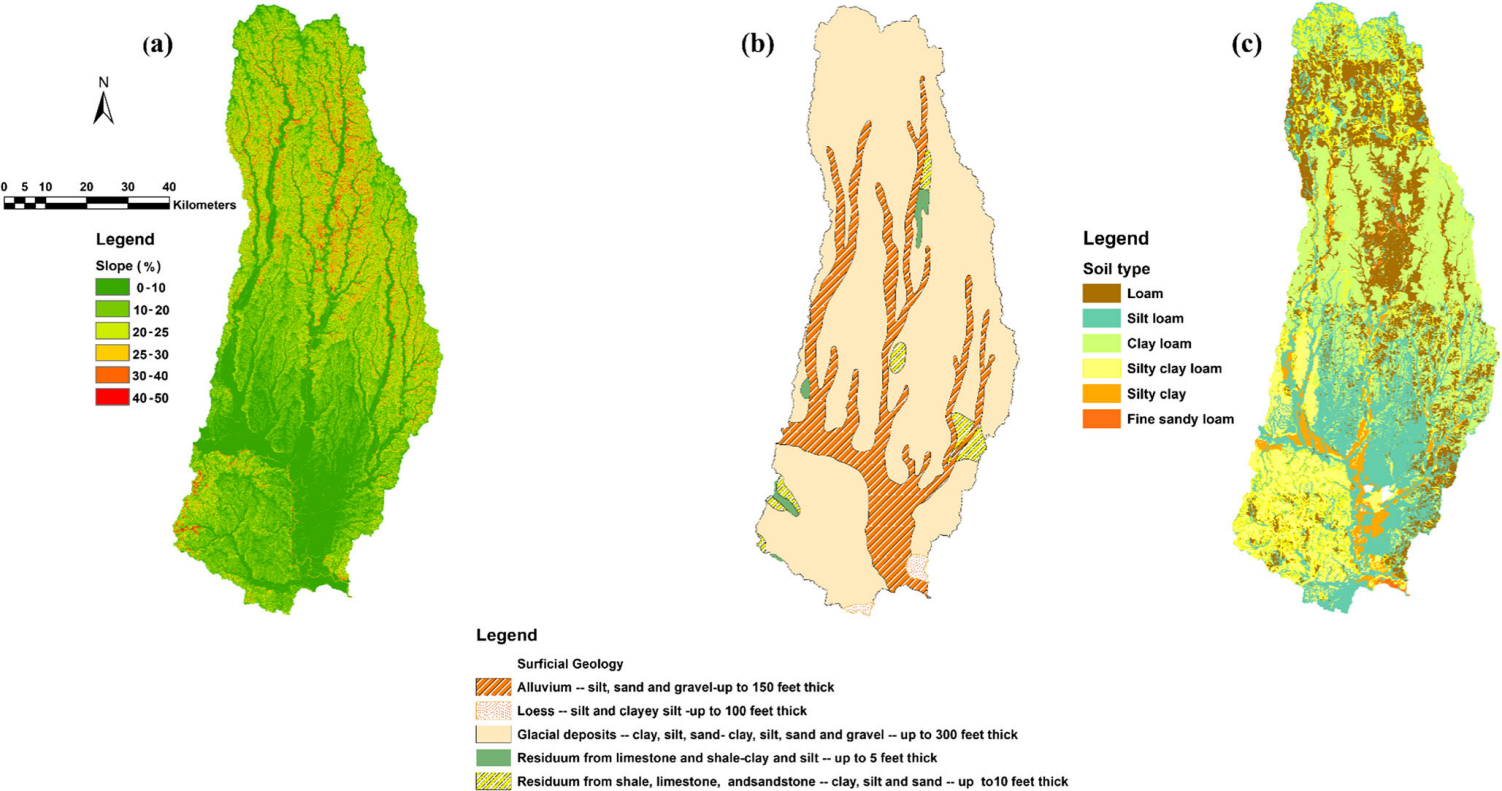
Characteristics of the Lower Grand River Watershed. **a** Percent slope. **b** Soil origin and thickness. **c** Soil texture

This study was conducted in the Lower Grand River Watershed, located in north-central Missouri and south-central Iowa (Fig. 1). The drainage area of the Lower Grand River Watershed is about 6112 km^2^, and the Grand River drains into the Missouri River as it exits this watershed. This watershed was chosen because it is representative, in terms of land use, geomorphology, and geologic characteristics, of many watersheds in the southern parts of the US Midwest. Thus, statistical correlations derived from this watershed may be applied to other regional watersheds with similar land use. The primary land use in the Lower Grand River Watershed is agricultural. About 48% of the watershed is used for pasture or hay, and 27% is used for cultivated crops, primarily corn, soybeans, and wheat. Approximately 13% percent of the land is forest, and 5% is urban. The topography of the Lower Grand River Watershed is fairly flat, with an average slope of 8°, as shown in Fig. 2a. Most of the study area is covered with Quaternary deposits of glacial drift and alluvium that are less than 30.5 m thick (Fig. 2b) (Gann et al. 1973). Soils in the study area are mostly loam, with loam, clay loam, and silt loam being the most common soil textures (Fig. 2c). Throughout the study area, the soils tend to be fertile and easily erodible (Detroy and Skelton 1983). The bedrock is primarily Pennsylvanian-age shale and limestone, with incised channels filled with sandstone (Vandike 1995).

According to the Midwestern Regional Climate Center (MRCC 2016), the average annual precipitation in the watershed ranges from 1029 mm in the north to 1054 mm in the south. The greatest volume of precipitation occurs in May and June, and stream discharge is highest during these months and lowest during the late summer and fall (USDA-SCS 1982). Since soil permeability is relatively low, most rainfall runs off into streams rather than infiltrating the groundwater, and streams typically exhibit rapid increases in discharge after precipitation, but quickly return to low flow conditions after surface runoff has stopped (MDNR 1984).

Surface water quality in the Lower Grand River Watershed is variable. According to Missouri Section 303(d), about 25% of the total length of the rivers and streams in the study area are listed as impaired (MDNR 2016). The most common types of known impairments are *Escherichia coli* (*E. coli*) contamination, high concentrations of phosphorus and nitrogen, high total suspended soils, and low DO (USEPA 2016; MDNR 2016). These impairments seem to be primarily a result of agricultural activities, although urban activities can also contribute to surface water degradation in the few watersheds with more development. Wilkison and Armstrong (2015) studied the impact of commercial fertilizers in the Lower Grand River Watershed, finding that the average application rates of agricultural chemicals, such as phosphorus and nitrogen, in this watershed have approximately doubled during the last four decades.

### Data acquisition and processing

**Fig. 3:**
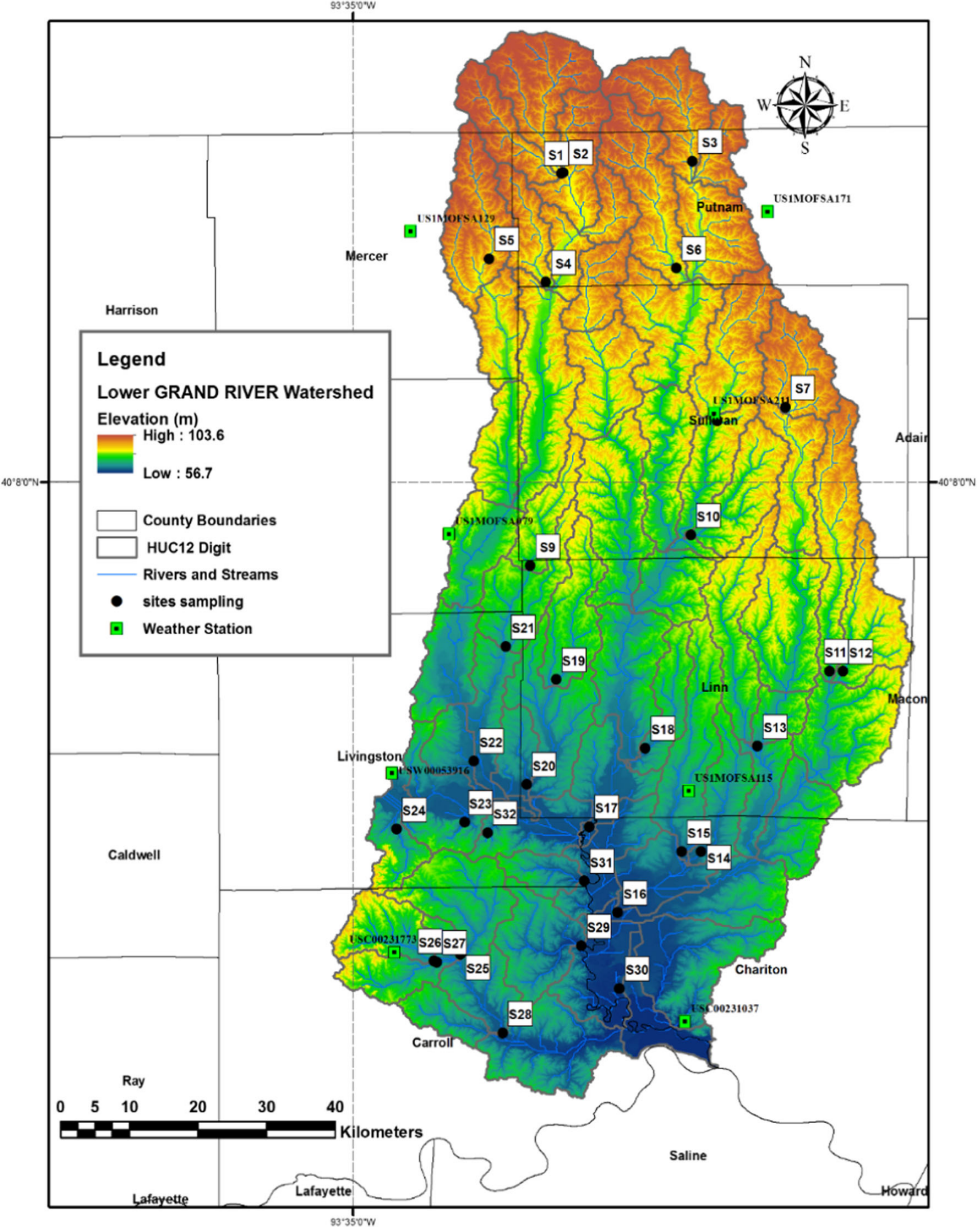
Map of the Lower Grand River Watershed showing HUC12-digit sub-watersheds, sampling locations, and precipitation stations

The Lower Grand River Watershed has been divided into 64 sub-watersheds, as defined by the US Geological Survey (USGS) hydrologic unit code HUC12-digit watersheds. Many of these sub-watersheds contain perennial streams that drain into the Grand River, although some sub-watersheds have intermittent streams (MDNR 2014). For this study, the geologic and LULC characteristics were determined for each of the 35 independent sub-watersheds in the Lower Grand basin, where an independent watershed is defined as one that receives no inflow from another watershed. Sampling was performed near the mouth of each sub-watershed (Fig. 3).

Surface water sampling was conducted in two major campaigns, in the late summer/fall of 2016 and spring of 2017, to monitor the streams after and before the primary growing season. For the late summer 2016 campaign, data were collected from 32 sub-watersheds over three weekends, August 3–4, September 11–12, and September 28–29. Three additional sub-watersheds were investigated, but the streams were dry. Relatively little precipitation occurred in the 2 weeks preceding data acquisition in the late summer/fall; the average precipitation in the 2 weeks preceding these campaigns was 1.87 mm (1.37 mm, 2.48 mm, and 1.75 mm, for the first, second, and third weekends, respectively). All precipitation measurements were calculated as the arithmetic average of the precipitation measured by eight rain gauges located within or adjacent to the study area, as shown in Fig. 3. Precipitation data were downloaded from the National Oceanic and Atmospheric Administration Climate Data database (NOAA 2017). In the spring 2017, data were acquired from 35 sub-watersheds on April 2–3 and April 9–10. More precipitation was received before the spring data collection; the average for the preceding 2 weeks before each campaign was 3.72 mm (2.74 and 4.71, for the first and second weekends, respectively). The stream discharge during each sampling campaign reflected the differences in precipitation. The average discharge of all the sampled streams during the late summer/fall was 3.6 m^3^/s, while the average discharge in the spring was 95 m^3^/s.

Although little precipitation occurred in the few weeks prior to data acquisition, the3 months of 2016 preceding the late summer/fall field campaign were approximately 26% wetter than average (i.e., average precipitation from July to September in 2006 through 2017 was 317 mm, while in 2016, it was 401 mm). This above-average precipitation may influence water quality by increasing baseflow above normal levels, although the streams monitored were mostly quite small and seemed more influenced by short-term (within the past few weeks) precipitation than by longer-term precipitation, as seen in the measured discharges. During the spring campaign, precipitation was close to average; average precipitation from February to April in 2006 through 2017 was 219 mm, while in 2017, the precipitation over these 3 months was 223 mm.

### GIS data processing

Data from remote sensing and field mapping techniques are available in a geographic information system (ArcGIS) database maintained by the Missouri Spatial Data Information Service (MSDIS) (n.d.). Figure 2 shows the slope, soil origin, and soil texture for the study area, as provided by the MSDIS. ArcGIS 10.2 was used to determine the values of the parameters for each of the 35 sub-watersheds. Some parameters, such as soil texture, LULC classification, depth to bedrock, depth to the water table, watershed area, and stream length, were obtained as shapefiles from the MSDIS. Other information, such as slope, topographic complexity, watershed shape index, watershed slope/relief ratio, and mean elevation, was derived from a 30-m resolution digital elevation model (DEM) provided by the MSDIS. ArcGIS was also used to analyze the data and to determine the average values of each parameter for each sub-watershed, as shown in Table 1.

**Table 1:** Minimum, maximum, mean, and standard deviation for independent variables

Variable	Description	Minimum	Maximum	Mean	Std. deviation
Area (km^2^)	Area of watershed	42.4	141.0	95.2	28.5
Watershed shape index	Area/square of watershed length	0.1	1.55	0.37	0.26
Average slope		1.97	7.28	4.35	1.51
Total stream length (km)	Total stream length in watershed	11.2	78.7	36.3	13.2
Topographic complexity	Standard deviation of elevation within watershed	12.90	47.7	28.9	11.2
Watershed slope/relief ratio (m/km)	Watershed elevation change/watershed length from outlet to highest point on perimeter	2.3	7.8	4.2	1.7
Mean elevation (m)	Mean elevation of watershed	215.7	306.3	250.1	23.8
Urban (%)	Percent of watershed	2.72	10.9	4.6	1.44
Forest (%)	Percent of watershed	3.2	28.90	12.4	5.60
Pasture/hay (%)	Percent of watershed	16.3	74.24	51.2	17.71
Cultivated crops (%)	Percent of watershed	3.6	66.9	24.9	16.5
Wetland (%)	Percent of watershed	0.34	23.5	4.1	6.3
Clay + silt (%)	Percent of clay and silt content	52.8	79.05	63.7	4.8
Average depth to groundwater (m)		3.05	11.7	7.17	2.01
Average depth to bedrock (m)		8.6	56.9	35.5	12.6
Discharge (m^3^/s) (measured in field)—fall		0.0085	0.95	0.16	0.22
Discharge (m^3^/s) (measured in field)—spring		0.81	23.94	2.7	4.36
Precipitation (mm) fall		0.00	19.05	2.46	5.83
Precipitation (mm) spring		45.7	92.4	65.8	19.8

**Fig. 4:**
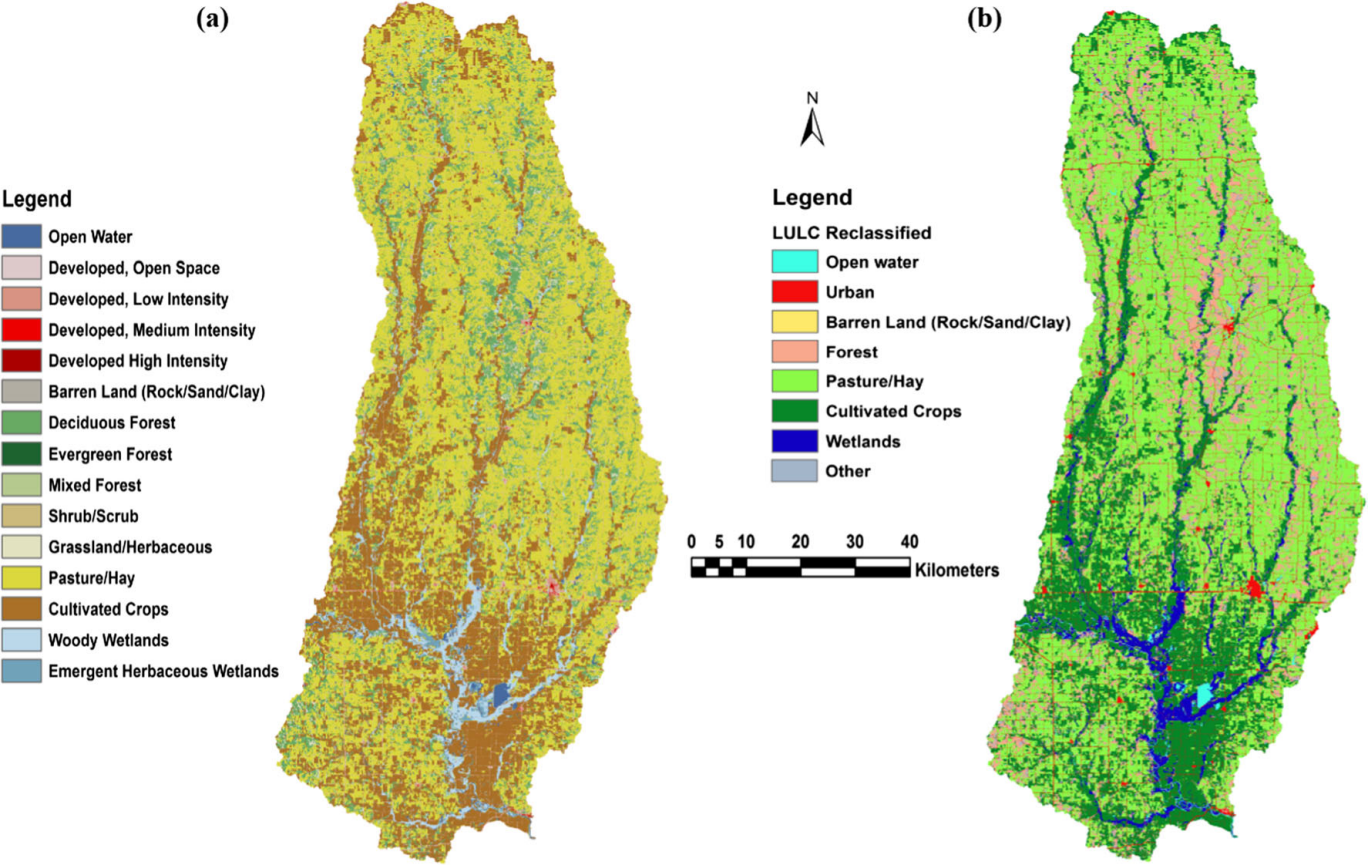
Land use categories **a** before reclassification and **b** after reclassification and aggregated into eight categories

LULC data were also analyzed using ArcGIS. The National Land Cover Database 2011 (Homer et al. 2015) includes 15 LULC categories (Fig. 4a). To reduce the number of independent variables and to create more meaningful LULC categories for this study, some of these categories were combined. All categories labeled “developed” were combined into one “urban” classification, and all categories labeled “forest” were combined into one group. Similarly, “wetland” categories were combined (Fig. 4b).

### Precipitation

To better understand how recent precipitation affects water quality parameters, the depth of precipitation was also estimated for each sub-watershed. To obtain the most accurate precipitation information, ground-based rain gauge data were used instead of satellite data. Precipitation depth was calculated as the sum of all precipitation that occurred in a 2-week period prior to data acquisition at the rain gauge station closest to each drainage basin. Since rain gauge data are not available for each sub-watershed, the precipitation value is an estimate based on the closest available data.

### Water quality parameters

#### Data acquisition

Surface water samples were collected from 32 sub-watersheds in August and September 2016 and from 35 sub-watersheds in April 2017. Fewer samples were collected in the fall 2016 because some streams were dry. Some water quality parameters were acquired in situ, including temperature, pH, SC, and DO, all of which were measured with a YSI ProPlus multimeter. Turbidity was also measured in the field using a Hach 2100Q portable turbidimeter. Samples were acquired in the field and tested for bacteria, phosphate (P), and nitrate (N) in the laboratory. All field measurements and samples were acquired using standard USGS procedures, including equipment calibration twice a day, cleansing of all equipment between samples, and following standard procedures to avoid contamination (USGS 2006). P and N samples were filtered on site and collected in sterilized polypropylene bottles. When needed, sulfuric acid was added to the N samples for preservation, if the samples could not be analyzed within 24 h of collection. Sample bottles were rinsed three times with stream water from the sampling sites before the samples were collected. Bacteria samples were collected in sterilized Whirl-Pak® bags. All samples were preserved on ice during transportation and refrigerated at 4 °C until they were processed. Bacteria samples were processed within 8 h of data collection, and N and P samples were processed within 24 h, except for a few N samples that were preserved with acid and processed within 48 h.

Laboratory procedures were based on manufacturers’ recommendations. Bacteria samples were processed using Coliscan® Easygel®, and samples were analyzed after 24 h of incubation for *E. coli* concentrations. N and P (orthophosphate) were analyzed using a Hach DR 3900 spectrophotometer. N concentrations were analyzed using the chromotropic acid method (Hach Method 10020), where N reacts with chromotropic acid to change the color of the solution, with a maximum absorbance at 410 nm. Soluble reactive P concentrations were analyzed using ascorbic acid (HACH standard procedure 8048). In this process, the P in the sample reacted with ammonium molybdate to form a phospho-molybdate complex, which then reacted with the ascorbic acid reagent to change the color of the solution. For both N and P, the concentrations were determined by measuring the intensity and wavelengths of light passing through the sample after reaction with the powder-pillow reagents.

Because water quality can change quickly with time, macroinvertebrate analysis was performed to assess the average water quality over a longer time period than was used for the water chemistry measurements (Paulsen et al. 2008; Buss and Vitorino 2010; Mereta et al. 2013; López-López and Sedeño-Díaz 2014; Van Ael et al. 2015; Gezie et al. 2017). Aquatic macroinvertebrates were acquired and identified using the bioassessment protocol for Missouri (MDNR 2003). The macroinvertebrates were collected using a 1000-μm kick net placed in the downstream section of a riffle zone. A 1-m by 1-m area immediately upstream of the net was disturbed by vigorous shuffling in the streambed. For sites that did not contain riffles, the net was placed downstream of a root mat, and the area around and underneath the root mat was disturbed. The net was then lifted, and macroinvertebrates were removed from the net, identified to the lowest taxonomic level (generally, genus), and counted. All remaining macroinvertebrates were placed into a sample jar and preserved with 80% ethyl alcohol for more rigorous identification in the laboratory. In general, macroinvertebrate collection was performed at two locations within each site. As macroinvertebrate collection at each site was very time-intensive, macroinvertebrates were acquired only during the fall 2016 and only at 16 sites.

Stream discharge was determined using standard USGS procedures. Each stream was divided into 20 evenly spaced intervals, and the water velocity and depth were measured at the center of each interval. A USGS Pygmy Meter Model 6205 was used to measure velocity. Stream discharge was calculated as the sum of the velocity, depth, and width for each interval, for all intervals of the product.

#### Summary of water quality parameters

To assess stream health based on macroinvertebrate populations, the biotic index (BI) was calculated (Eq. 1). The BI is based on the classification of macroinvertebrates depending on their tolerance of pollution and was calculated for each site using

1 $$ BI=\sum \limits_{i=1}^s\frac{T{V}_i{N}_i}{N_t} $$

where *S* is the number of taxa in the sample, *TV*_*i*_ is the pollution tolerance value of the *i*th taxon, *N*_*i*_ is the density of the *i*th species taxon as abundance (numbers per square meter), and *N*_*t*_ is the total number of macroinvertebrates in the sample (Lenat 1993). Tolerance values range from 0 (highly intolerant) to 10 (highly tolerant) and were chosen for each taxon using the protocol developed by Sarver (2005), which is applicable to this study area. The BI is also scored from 0 to 10 (Table 2), with 0 indicating generally excellent water quality and 10 indicating generally very poor water quality (Hilsenhoff 1988).

**Table 2:** Biotic index and pollution levels

Biotic index	Water quality rating	Degree of organic pollution
0.00–3.5	Excellent	No apparent organic pollution
3.51–4.5	Very good	Slight organic pollution possible
4.51–5.5	Good	Some organic pollution probable
5.51–6.5	Fair	Fairly substantial pollution likely
6.51–7.5	Fairly poor	Substantial pollution likely
7.51–8.5	Poor	Very substantial pollution likely
8.51–10.0	Very poor	Severe organic pollution likely

Stream health can also be assessed using the water quality index (WQI) (Eq. 2), which was calculated using the method developed by Cude (2001). The WQI is based on the sub-index measurements of pH, temperature, DO, biochemical oxygen demand, nitrate, total phosphorus, total dissolved solids, and fecal coliform. It provides a summary of water quality, ranging from 0 (very poor) to 100 (excellent) (Kaurish and Younos 2007; Ramos et al. 2016).

2 $$ WQI=\sum \limits_{i=1}^nS{I}_i{W}_i $$

where *WQI* is the water quality index, *SI* is sub-index *i*, and W_*i*_ is the weight given to sub-index *i.*

#### Statistical data analysis

Statistical analyses were performed using the Statistical Package for Social Sciences (SPSS) software. The water quality parameters were first analyzed using the Cunnane probability method to determine if they were normally distributed at α = 0.01. The critical correlation coefficients for the fall (*n* = 32) and spring (*n* = 35) data sets were 0.950 and 0.954, respectively. Some factors were normally distributed without any transformations, but others required transformation. Various transforms were tried (e.g., logarithmic, natural log, square root, and cubed root), and the transform with the highest correlation coefficient (*R*) (closest to the normal distribution) was applied in all further analyses. If the data were normally distributed without a transformation, no transformation was performed. All parameters were normally distributed either before or after transformation.

Six analyses were performed on the water quality data. First, the standard parametric summary statistics were calculated for each variable. Next, a pairwise comparison was performed for each water quality variable acquired in the spring and fall. The differences for each characteristic were calculated, and the Cunnane method was again employed to determine whether the differences were normally distributed. If the differences were normal, the paired *t* test was employed to determine if the two data sets were statistically different. If the differences were not normal, the sign test was used. The third analysis was a simple linear regression between each independent variable (i.e., LULC, geologic, or topographic parameters) and each dependent variable (i.e., water quality parameter) to determine the strength and direction of the correlation between each pair of variables. The fourth analysis was a stepwise linear regression to determine which independent variables were most useful for predicting water quality parameters. The partial F entry test and partial F removal test had a significance level of α = 0.05. The coefficient of multiple determination (*R*^2^) for each regression equation indicates the proportion of the variability in the water quality parameters that can be explained by the independent variable. The fifth analysis compared the biotic index values with the WQI to determine how well the biotic index predicted the WQI. The final analysis was a principal component analysis of the physicochemical water quality variables and the BI.

## Results

### Summary statistics of water quality parameters

Summary statistics for each of the water quality parameters measured in this experiment are shown in Table 3. This table shows that significant variations in water quality occurred between watersheds within each data campaign and that some parameters varied significantly between data campaigns. Temperature was much higher during the fall than during the spring, which indicates that the streams probably had a larger proportion of surface runoff compared to baseflow during the fall. Temperature was also more variable during the fall, which may be related to the generally lower discharge during this season, as smaller streams are more susceptible to changes in air temperature. Two of the least variable parameters were pH and P, with relatively little variation between watersheds or with season. SC showed significant variations between watersheds, but relatively little variation with season. DO was significantly higher during the spring, perhaps due to increased turbulence in the streams, associated with higher discharge. Turbidity, N, and *E. coli* counts, all of which would be expected to increase with increasing overland flow, had much higher values during the spring.

**Table 3:** Summary statistics of water quality parameters for two sampling campaigns

Variable	Fall				Spring			
	Minimum	Maximum	Mean	Std. deviation	Minimum	Maximum	Mean	Std. deviation
T (°C)	16.10	28.60	21.55	3.62	10.10	15.40	12.3	1.53
pH	7.13	8.35	7.77	0.40	7.65	8.75	8.26	0.32
DO (mg/L)	0.30	9.51	3.48	2.38	4.65	11.18	9.10	1.85
SC (μs/cm)	205.60	605.00	307.34	99.28	150.00	461.90	271.74	78.84
Turbidity (NTU)	4.33	219.00	47.64	54.59	17.50	428.00	94.88	89.5
Phosphate (mg/L)	0.12	13.43	1.12	3.28	0.19	10.38	0.74	1.70
Nitrate (mg/L)	0.10	21.60	1.77	5.29	0.64	18.80	2.78	3.16
*E. coli* (cfu/100 mL)	100.0	1350.0	509.37	347.47	0.00	4550.00	1012.85	1245.78
Biotic index (BI)	4.0	7.42	5.35	1.02				
WQI	51.63	84.65	66.30	8.43	42.67	85.56	68.73	8.86

### Pairwise comparison of fall and spring data

Table 4 shows the pairwise comparisons for each water quality parameter that was acquired in both the fall and spring. The fall and spring data sets were statistically different, with fairly low *p* values for all water quality parameters. This suggests that temporally variable factors influencing these parameters may be more important than static factors in estimating surface water quality.

**Table 4:** Normality test results and pairwise comparison of fall and spring data sets

Parameter	Fall: normal without transform (*R*)	Fall: best transform	Fall: normal after transform (*R*)	Spring: normal without transform (*R*)	Spring: best transform	Spring: normal after transform (*R*)	Differences between fall and spring normally distributed (*R*)	Statistical method employed	Statistically different in spring and fall (*p* values)
Temperature	Yes (0.991)	Square root	Yes (0.999)	Yes (0.974)	Square root	Yes (0.999)	Yes (0.986)	Paired-t test	Yes (< 0.001)
pH	Yes (0.994)	Square root	Yes (0.999)	Yes (0.969)	Square root	Yes (0.999)	Yes (0.96)	Paired-t test	Yes (< 0.001)
SC	Yes (0.959)	Square root	Yes (0.995)	Yes (0.982)	Square root	Yes (0.997)	Yes (0.97)	Paired-t test	Yes (0.013)
DO	Yes (0.995)	Square root	Yes (0.971)	Yes (0.994)	Square root	Yes (0.998)	Yes (0.98)	Paired-t test	Yes (< 0.001)
Turbidity	No (0.667)	Square root	Yes (0.969)	No (0.827)	Square root	Yes (0.979)	No (0.89)	Sign test	Yes (0.002)
Nitrate	No (0.444)	Square root	Yes (0.962)	No (0.713)	Square root	Yes (0.968)	No (0.92)	Sign test	Yes (< 0.001)
Phosphate	No (0.516)	Square root	Yes (0.961)	No (0.512)	Square root	Yes (0.970)	No (0.68)	Sign test	Yes (0.011)
*E. coli*	No (0.884)	Square root	Yes (0.950)	No (0.868)	Square root	Yes (0.971)	No (0.92)	Sign test	Yes (0.016)
Biotic index	Yes (0.973)	Square root	Yes (0.993)	NA	NA	NA	NA	NA	NA

### Simple regression

Simple regression analysis was done between all water quality indicator variables and all independent variables (i.e., LULC, geologic, and topographic factors). For water quality characteristics that were not normal before transformation (i.e., turbidity, N, P, and *E. coli*), the transformed (square root) data were used for the correlation analysis. The correlation coefficient (Pearson’s coefficient or *R*) and the statistical significance of each regression relationship is shown for the most significant correlations between water quality variables and the independent variables in Tables 5 and 6 for the fall and spring, respectively. These tables illustrate that the independent variables that best correlate with water quality indicators vary with season for some water quality indicators but remain more temporally consistent with others. During the fall, the independent variable that correlated most often with water quality was the “pasture/hay” land use category; this land use was significant for N, P, *E. coli*, and turbidity. Since pasture includes land where livestock graze, it is probable that these water quality parameters are affected by animal waste and/or erosion created by animals near streambanks (Walters et al. 2011). The percent of urban land also correlated with multiple water quality parameters, including *E. coli*, P, and temperature. The Lower Grand watershed is predominantly rural, but several sub-watersheds include developed areas. Leaching from septic tanks, municipal sewage, lawn fertilizers or urban stormwater runoff may impact streams. Although the fall was relatively dry, the second most frequently observed independent variable was precipitation, which was the most significant factor related to N and SC. These correlations suggest that even small amounts of precipitation can be significant for transporting nutrients and other dissolved solids to surface water (Narasimhan et al. 2010; Jeznach et al. 2017). DO correlated best with the geologic factors of depth to bedrock and depth to groundwater, while temperature and pH had only weak or statistically insignificant correlations.

**Table 5:** Correlation coefficients between water quality indicators and watershed landscape characteristics during the fall

Factor of correlation	*R*	*p* value	Factor of correlation	*R*	*p* value	Factor of correlation	*R*	*p* value
DO			pH			Temperature		
Average depth to bedrock (m)	0.72	0.000	Discharge (m^3^/s)	− 0.15	0.25	Urban%	0.53	0.05
Average depth to groundwater (m)	0.52	0.006						
SC			*Escherichia coli* (*E. coli*)			Turbidity		
Precipitation (mm)	− 0.47	0.012	Urban%	0.37	0.045	Clay + silt%	0.63	0.000
			Pasture/hay%	0.37	0.05	Pasture/hay%	0.58	0.005
						Average slope	0.54	0.001
Nitrate			Phosphate			Biotic index (BI)		
Precipitation (mm)	0.6	0.013	Urban%	0.4	0.031	Turbidity (NTU)	0.58	0.008
Pasture/hay%	0.40	0.03	Pasture/hay%	0.33	0.03	Phosphate (mg/L)	0.47	0.031

**Table 6:** Correlation coefficients between water quality indicators and watershed landscape characteristics during the spring

Factor of correlation	*R*	*p* value	Factor of correlation	*R*	*p* value	Factor of correlation	*R*	*p* value
DO			pH			Temperature		
Average depth to groundwater (m)	0.55	0.000	Average depth to groundwater (m)	0.60	0.000	Pasture/hay%	0.62	0.000
Precipitation (mm)	0.30	0.040	Clay + silt%	0.47	0.02	Cultivated crops%	0.60	0.000
SC			*Escherichia coli* (*E. coli*)			Turbidity		
Average slope	0.70	0.000	Urban%	0.41	0.003	Discharge (m^3^/s)	0.50	0.001
Average depth to bedrock (m)	− 0.55	0.000	Pasture/hay%	0.3	0.043	Average slope	0.37	0.013
Cultivated crops%	0.54	0.000						
Nitrate			Phosphate			Biotic index		
Pasture/hay%	0.40	0.012	Pasture/hay%	0.43	0.031	Nitrate (mg/L)	0.52	0.019
Cultivated crops%	0.30	0.020	Precipitation (mm)	0.40	0.040	Phosphate (mg/L)	0.45	0.040
						Turbidity (NTU)	0.30	0.012

The spring data exhibited many of the same independent factors correlated to water quality parameters along with several new correlations. Unlike in the fall, cultivated crops had more effect, being significantly correlated with N, SC, and temperature. This effect might result from the timing of fertilizer application because approximately twice as much fertilizer is applied near planting time in the spring than during the fall in Missouri (Fulhage 2000; Missouri Agricultural Experiment Station 2014). The composition of the fertilizer is also significant, as approximately four times as much nitrogen is applied in the spring as in the fall, but the amount of phosphatic fertilizer is approximately equal in the spring and fall (Missouri Agricultural Experiment Station 2014). The percentage of land classified as urban was less significant during the spring, when only *E. coli* correlated with this parameter.

An evaluation of regression coefficients indicates that only some of the factors most highly correlated with water quality indicators are seasonal. This variability is probably due to changes in the proportion of surface runoff and baseflow in streams. Geologic factors, such as depth to groundwater and slope as well as LULC factors correlated strongly with water quality indicators. This means that topographic and geologic factors cannot be neglected when determining the watersheds with the greatest risk of water quality impairment.

#### Stepwise multiple regression

Stepwise multiple regression was performed to determine which independent variables were most suitable for predicting water quality indicators in different seasons. Stepwise regression only employs independent variables that significantly improve the correlation after other independent variables are considered. For example, slope and topographic complexity may both correlate strongly with water quality, but these independent variables are often correlated. Therefore, it is not useful to include them both in a regression equation because it would not greatly improve the estimation of a water quality indicator. In addition, it would add unnecessary complexity to the relationship and make data acquisition more arduous. Consequently, the only parameters included in the following stepwise regression equations are those that most significantly and independently improve the correlation to water quality indicators. As with the correlation analysis, water quality parameters that were not normal before transformation were transformed prior to regression, but those that were normally distributed without a transformation were not transformed. Table 7 displays the stepwise regression results for the fall, while Table 8 presents similar results for the spring.

**Table 7:** Stepwise regression models between water quality indicators and watershed landscape characteristics during the fall

	Beta coefficients	*R*	*R* ^2^	*p* value
Model for temperature				
Average depth to bedrock	− 0.07	0.84	0.70	0.000
Total stream length	0.13			
Beta coefficients (constant) = 26.4				
Regression equation: Temperature = 26.4–0.07 (average depth to bedrock) + 0.13 (total stream length)				
Models for *E. coli*				
Urban	3.6	0.56	0.32	0.006
Beta coefficients (constant) = − 10.4				
Regression equation: *E. coli* = 3.6 (urban) − 10.4				
Model for pH				
Precipitation	− 0.18	0.32	0.10	0.000
Beta coefficients (constant) = 8.44				
Regression equation: pH = 8.44–0.18 (precipitation)				
Model for DO				
Average depth to bedrock	0.04	0.72	0.52	0.007
Average depth to groundwater	0.1			
Beta coefficients (constant) = −3.2				
Regression equation: DO = − 3.2 + 0.04 (average depth to bedrock) + 0.1 (average depth to groundwater)				
Model of turbidity				
Average slope	− 0.25	0.64	0.4	0.002
Urban	− 3.41			
Beta coefficients (constant) = 119.7				
Regression equation: Turbidity = 119.7 − –0.25 (average slope) − 3.41 (urban)				
Model of SC				
Precipitation	11.06	0.83	0.70	0.002
Clay + silt	4.3			
Beta coefficients (constant) = − 309.4				
Regression equation: SC = − 341.73 + 11.06 (precipitation) + 4.3 (clay + silt)				
Model for nitrate				
Precipitation	0.46	0.53	0.28	0.001
Urban	0.37			
Beta coefficients (constant) = − 1.1				
Regression equation: Nitrate = 0.46 (precipitation) + 0.37 (urban) − 1.1				
Model for phosphate				
Precipitation	0.07	0.57	0.32	0.02
Beta coefficients (constant) = 0.57				
Regression equation: Phosphate = 0.57 + 0.07 (precipitation)				
Model for biotic index (BI)				
Turbidity	0.3	0.88	0.78	0.002
Urban	− 0.9			
Temperature	0.14			
Beta coefficients (constant) = 4.25				
Regression equation: BI = 0.3 (turbidity) − 0.9 (urban) + 0.14 (temperature) + 4.25

**Table 8:** The stepwise regression models between water quality indicators and watershed landscape characteristics during the spring

	Beta coefficients	*R*	*R* ^2^	*p* value
Model for temperature				
Average slope	1.2	0.78	0.61	0.000
Watershed slope/relief ratio	− 0.57			
Average depth to bedrock	− 0.01			
Beta coefficients (constant) = 11.8				
Regression equation: Temperature = 11.8 + 1.2 (average slope) − 0.57 (watershed slope/relief ratio) − 0.01 (average depth to bedrock)				
Model for *E. coli*				
Urban	4.3	0.60	0.36	0.001
Beta coefficients (constant) = 24.5				
Regression equation: *E. coli* = 4.3 (urban) + 24.5				
Model for pH				
Average depth to groundwater	0.03	0.67	0.46	0.002
Precipitation	0.005			
Beta coefficients (constant) = 7.03				
Regression equation: pH = 7.03 + 0.03 (average depth to groundwater) + 0.005 (precipitation)				
Model for DO				
Average depth to groundwater	0.15	0.55	0.30	0.001
Beta coefficients (constant) = 5.42				
Regression equation: DO = 0.15 (average depth to groundwater) + 5.42				
Model of turbidity				
Discharge	0.011	0.61	0.37	0.001
Average slope	− 0.12			
Beta coefficients (constant) = 11.35				
Regression equation: Turbidity = 0.011 (discharge) − 0.12(average slope) + 11.35				
Model of SC				
Average slope	29.6	0.75	0.57	0.001
Average depth to bedrock	0.5			
Beta coefficients (constant) = 82.6				
Regression equation: SC = 29.6 (average slope) + 0.5 (average depth to bedrock) + 82.6				
Model for nitrate				
Pasture/hay	−0.02	0.43	0.18	0.053
Average slope	0.14			
Beta coefficients (constant) = 3.03				
Regression equation: Nitrate = 0.014 (average slope) − 0.02 (pasture/hay) + 3.03				
Model for phosphate				
Average slope	0.21	0.51	0.26	0.024
Urban	0.08			
Beta coefficients (constant) = 3.47				
Regression equation: phosphate = 0.21 (average slope) + 0.08 (urban) + 3.47				
Model for biotic index				
Nitrate	0.86	0.67	0.45	0.037
Precipitation	−0.02			
Beta coefficients (constant) = 5.5				
Regression equation: BI = 0.86 (nitrate) − 0.02 (precipitation) + 5.5				

Table 7 shows that during the fall, a statistically significant regression equation could be generated for each of the water quality indicators, but the quality of these predictions (as shown by the *R*^2^ value) was often low. The parameters where more than 50% of the variance could be predicted using regression relationships were temperature, DO, SC, and biotic index. In some cases, the independent variables in the regression equation were the same as those with high correlation coefficients in Table 5; however, other water quality indicators were best predicted by variables without the highest correlation. For the stepwise regression relationships with higher Pearson coefficients, geologic parameters (e.g., depth to bedrock, depth to groundwater, soil type) were often more helpful for predicting water quality indicators than were LULC characteristics. For several of the relationships with lower Pearson coefficients, precipitation was the most significant variable, suggesting that the timing of a measurement may strongly influence the result.

During the spring (Table 8), the regression relationships often had lower Pearson coefficients than during the fall. Only temperature and SC had relationships where more than 50% of the variability could be explained by the correlation variables. As with the fall, geologic or topographic parameters had a greater effect than LULC variables, although urban land use was significant for *E. coli* and P, and pasture/hay was important for N.

A comparison of stepwise regression relationships developed using data acquired during the spring and fall show that for approximately half of the water quality parameters (e.g., temperature, *E. coli*, pH, DO, and turbidity), one independent variable occurs in the regression equation for both seasons. However, the relationships developed using the spring data present differing (usually additional) independent variables. The independent variable that remains significant across both seasons tends to be the most critical predictor for each water quality indicator. For some water quality indicators, such as SC, N, and P, the independent variables in the regression relationships differ completely depending on season. This suggests that the loading mechanisms for these parameters may vary significantly with season and recent land use modifications, such as fertilizer application, so different seasonal models may be required to predict water quality using simple stepwise regression relationships.

#### Water quality and biotic indexes

**Fig. 5:**
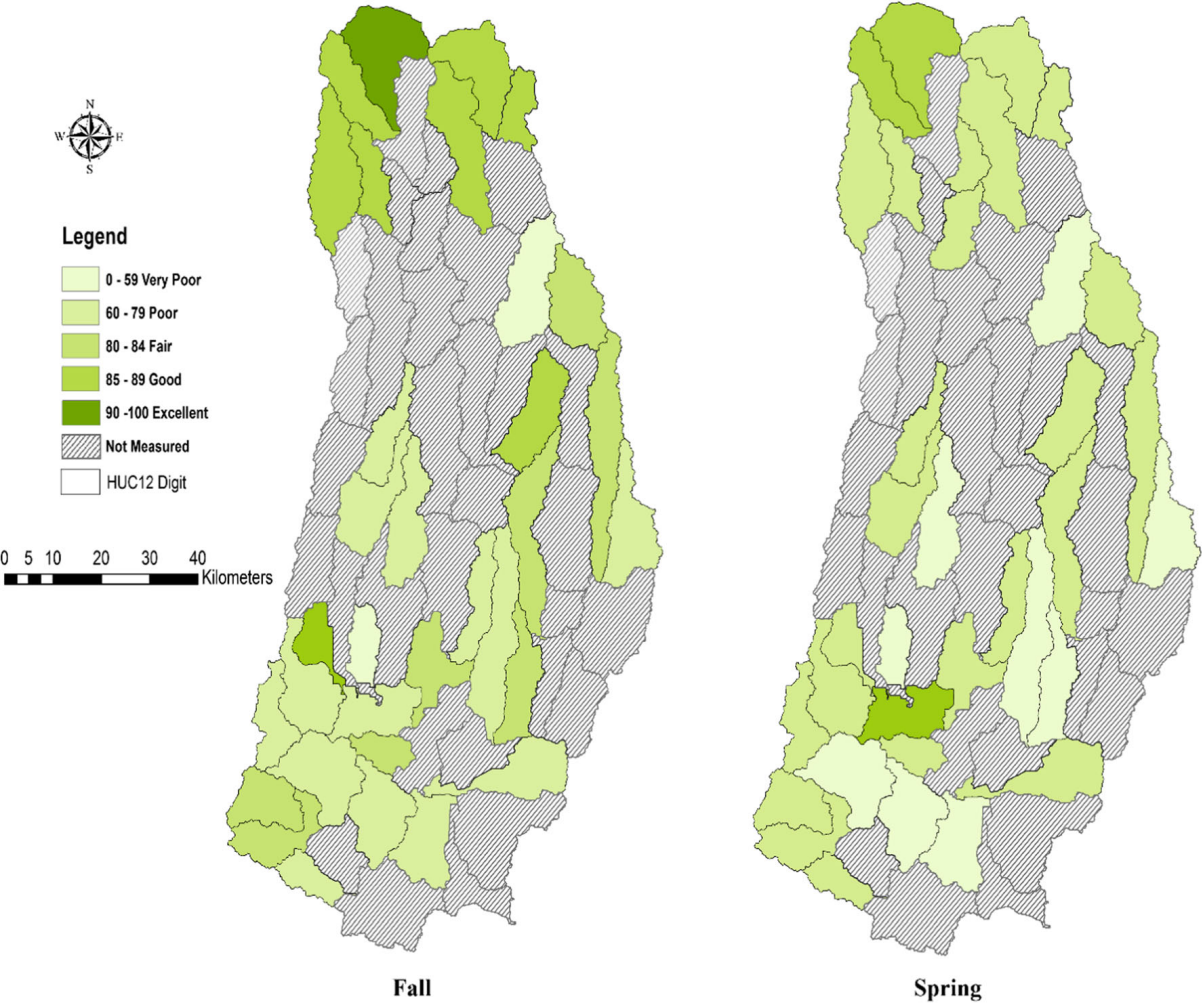
Spatial distribution of the WQI for the study area during the fall and spring

The results of the WQI are shown in Fig. 5. The fall WQI values ranged from 52 (very poor) to 97 (excellent), while WQI values during the spring ranged from 43 (very poor) to 86 (very good). During the spring, about 70% of the watershed sites were degraded. The lower WQI in the spring might have been caused by increased surface runoff that carried recently applied nutrients, sediment, and bacteria to the streams.

**Fig. 6:**
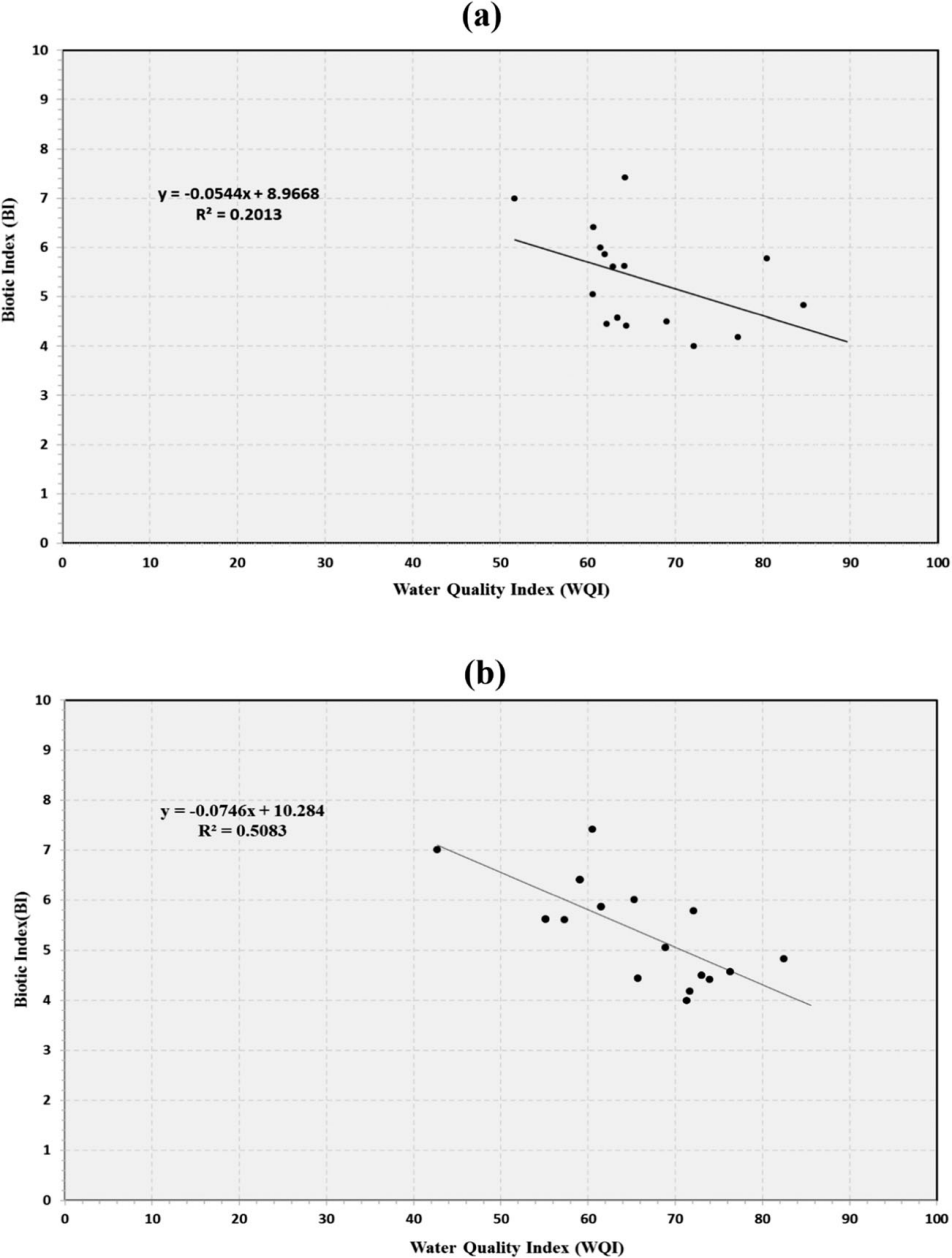
Comparison between the water quality index (WQI) and biotic index (BI). **a** Fall. **b** Spring

The WQI value is based on several physicochemical water quality parameters and bacterial concentration. These parameters may change with time and are difficult to measure on a continuous basis. Macroinvertebrate populations are more time-consuming to sample in the field but can provide information about average water quality over time. Figure 6a compares the WQI and biotic index for the fall data, displaying the expected trend between these variables; however, the correlation is too low to meaningfully relate these two parameters. Figure 6b presents the biotic index data acquired in the fall with the WQI calculated using water quality measurements collected in the spring. Even though these data sets were acquired at different times, there is a significantly better correlation between the WQI and the biotic index for the spring measurements than for the fall. This suggests that the water quality measurements acquired in the spring may be more indicative of the longer-term conditions for the streams in this study.

#### Principal component analysis

**Fig. 7:**
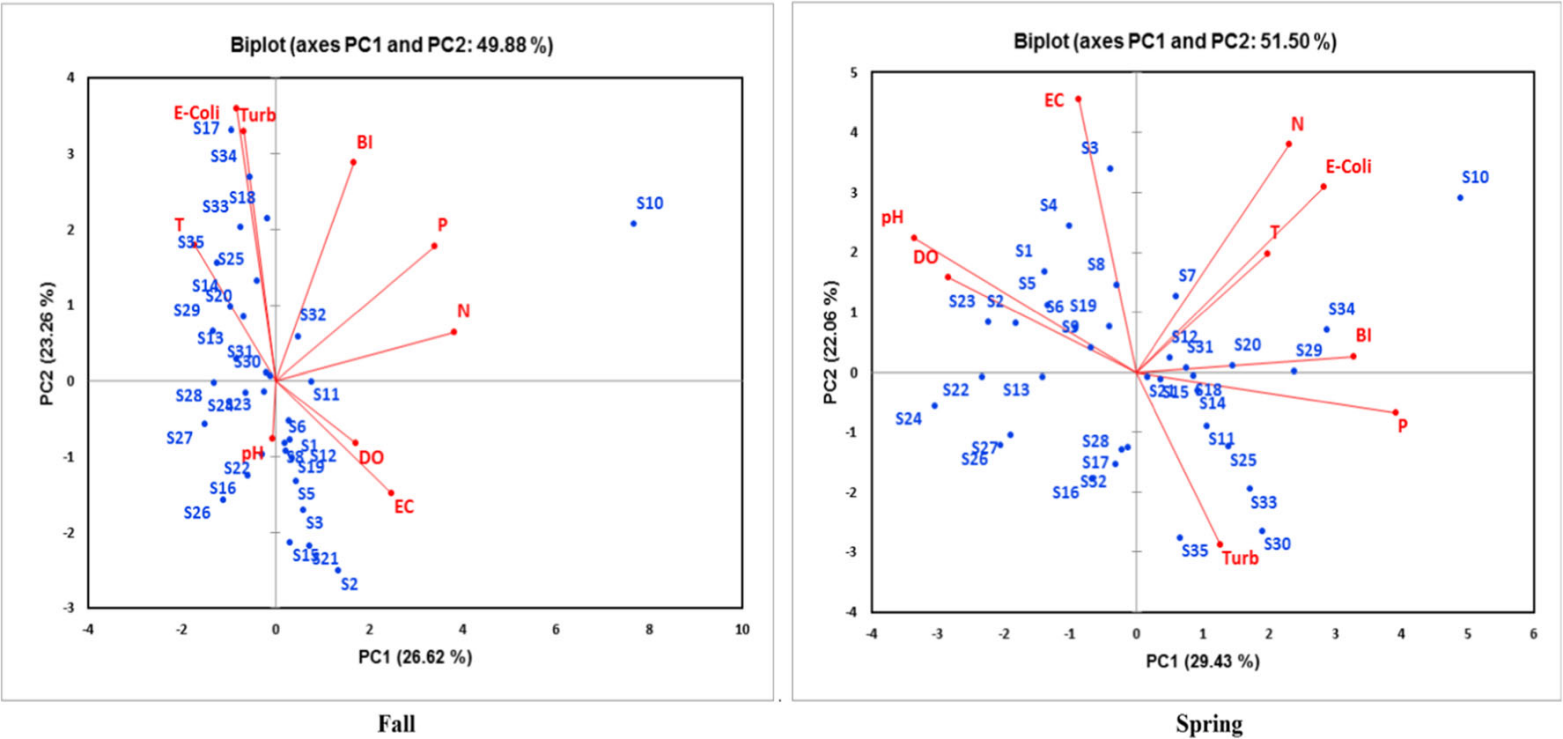
PCA biplots of water quality indicators for fall and spring based on the first two PCs

Three principal components were obtained with eigenvalues > 1, which accounted for 68.4% of the total variance in the data set in the fall and 69.2% in the spring. Figure 7 illustrates the first two principal components for each of these seasons, while Table 9 presents the strength of the correlation for individual parameters.

**Table 9:** Factor loadings values of water quality indicators for fall and spring

	Fall			Spring		
	PC1	PC2	PC3	PC1	PC2	PC3
Parameter						
T	− 0.411	0.397	− 0.646	0.400	0.346	0.693
pH	− 0.012	− 0.171	0.465	− 0.678	0.393	− 0.313
DO	0.411	− 0.185	0.727	− 0.574	0.276	− 0.342
EC	0.591	− 0.330	− 0.431	− 0.176	0.796	0.507
Turbidity	− 0.195	0.800	0.311	0.255	− 0.503	− 0.302
P	0.810	0.396	− 0.201	0.790	− 0.117	− 0.137
N	0.912	0.142	− 0.246	0.465	0.664	− 0.476
*E. coli*	− 0.159	0.732	− 0.038	0.571	0.540	− 0.529
BI	0.398	0.641	0.346	0.662	0.045	0.169
Eigenvalue	2.396	2.094	1.668	2.649	1.986	1.596
Total variance (%)	26.61	23.26	18.52	29.43	22.06	17.73
Cumulative variance (%)	26.61	49.88	68.41	29.43	51.49	69.23

In the fall, the first principal component (PC1) correlated most highly with P and N, and more weakly with SC. This component seems to be primarily associated with fertilizer runoff. The second principal component (PC2) correlated most highly with turbidity, *E. coli*, and BI. Turbidity may be affected by manure application but may also be strongly influenced by grazing livestock and associated streambed erosion. The correlations observed in PC2 imply that the biotic index could be more affected by livestock-related runoff (either directly from grazing livestock or from manure application to fields) than by the application of chemical fertilizers. In the spring, parameters were more similarly correlated with both PC1 and PC2, with fewer very strong correlations with either component than in the fall. PC1 was most correlated with P, pH, and BI, while PC2 was most correlated with SC and N. Since the BI data were only acquired in the fall, the apparent correlation between BI and P in the spring (Fig. 7) may not be significant. However, the correlation between N and *E. coli* in the spring may indicate a common livestock-based source for these factors.

## Discussion

The results of this study reveal that water quality parameters can vary significantly with season and may reflect recent land use, such as fertilizer application. Many of the results followed expected patterns; DO and turbidity are both higher when discharge is larger (i.e., in the spring, in this study). SC was lower during the spring, perhaps due to dilution. *P*-values were higher in the fall. This can be explained by higher discharge in the spring even though fertilizers are applied in approximately equal amounts in the fall and spring. N and *E. coli* are significantly higher in the spring, when more nitrogen-based fertilizer is applied and when more manure may also be applied.

Compared to the literature, our study found similar results in its correlations of water quality with land use, geologic, or topographic parameters. For example, Tong and Chen (2002) studied correlations between land use and water quality parameters in watersheds in Ohio. They used data available from the US Environmental Protection Agency (USEPA) averaged over an 8t-year period and found that nitrogen, phosphorus, and fecal coliform were all positively correlated with both agricultural and urban land use. Similarly, our research found that these water quality parameters were correlated with pasture/hay land use, and *E. coli* and P were also correlated with the percentage of urban land. During the spring, cultivated crops were also significant for N. The correlation analysis (Spearman’s rank) performed by Tong and Chen (2002) showed that the correlations between each of these water quality parameters and urban land use was greater than the correlation with agricultural land use. Even though the percent of urban land in our study was small, our results also established that the percent of urban land was significant, although not always more significant than agricultural land use. The correlation factors (i.e., Pearson’s correlation coefficient) in our investigation were generally higher than those observed by Tong and Chen (2002), possibly because we collected data for a relatively short time, whereas their data over a longer time span.

Galbraith and Burns (2006) focused on the impact of land modification on water quality in non-flowing water bodies (e.g., lakes, wetlands, estuaries, etc.) in southern New Zealand. They found that the conversion of native grasslands to pasture increased nutrient concentrations and turbidity. The Lower Grand study also showed that pasture/hay land use was highly correlated to nutrient concentrations and turbidity as well as to *E. coli*.

The results of this study were less similar to research conducted in the eastern USA, which has a very different physiography. Potter et al. (2005) considered the impact of land use as well as of topographic and geologic factors on benthic macroinvertebrates in North Carolina, and they found that forest was the land use variable that correlated most closely with macroinvertebrate health, while watershed shape was the second most important variable. However, we found that neither of these variables showed a high correlation with macroinvertebrate health, possibly because we studied primarily agricultural watersheds, not those what were heavily forested. Also, our study correlated chemical water quality parameters with macroinvertebrate health, with nutrients and turbidity being highly correlated to the biotic index.

On the east coast, Schoonover and Lockaby (2006) studied the impact of land cover in 18 watersheds in western Georgia. The watersheds in their study were much more urbanized than the Lower Grand River watersheds, and row crops were rare. Most watersheds in their study area were dominated by a single land cover class (i.e., unmanaged forest, managed forest, pasture, developing, or urban). They found that more urbanized watersheds typically had higher nutrients and *E. coli* than less urbanized watersheds. In the Lower Grand watershed, the percentage of land classified as urban is small, but urban land use still occurred as a factor that correlated significantly with several water quality parameters. This suggests that runoff from developed land, septic tanks, or municipal sewage may significantly impact water quality even in areas that are predominantly rural. Schoonover and Lockaby’s (2006) work also had a temporal component. They found that nutrient concentrations were higher during storm flow than during baseflow conditions. In the Lower Grand study, nutrient concentrations seemed to be more influenced by the timing of fertilizer application. As such, concentrations of N were significantly higher in the spring (when more nitrogen fertilizer is applied) than in the fall. P concentrations were higher in the fall, even though P fertilizer is applied in approximately equal amounts in the spring and fall.

PCA analysis demonstrated significant seasonal variations in PC1 and PC2 factors, as did other studies (Ouyang et al. 2006; Garizi et al. 2011). Several of the factors that influenced variability in the fall were the same as those observed by other researchers. Ouyang et al. (2006) acquired data in the fall and spring along the lower St. John’s River in Florida, and they found that the most influential parameters for PC1 were N, P, and EC (related to SC) (positively correlated) and organic carbon (negatively correlated). In another study along the Nakdong River, Jung et al. (2016) discovered that PC1 was influenced by N, P, EC, organic carbon, and chemical oxygen demand. In the Lower Grand River, the fall PC1 was most influenced by N, P, and SC (positively correlated). In the spring, Ouyang et al. (2006) found that PC1 was most influenced by color, organic carbon (positively correlated) as well as alkalinity and SC (negatively correlated), while our study found that SC was weakly negative correlated with PC1 but strongly and positively correlated with PC2 in the spring.

## Conclusions

Basic water quality measurements were acquired in 35 primarily agricultural watersheds during the fall and following spring. These measurements were used to calculate the biotic index and water quality index and were correlated with a variety of geologic, topographic, and LULC parameters. Pairwise comparison of the data acquired during the fall and spring showed that all water quality parameters were statistically different data sets with *p* < 0.02 for all parameters, which suggests that the timing of water quality sampling is critical. Simple regression analysis of all variables revealed that correlations between independent variables and water quality indicators fluctuated with the season but that the “pasture/hay” LULC category (which includes livestock grazing) was statistically significant for several water quality indicators for both sampling campaigns. The percentage of land used for cultivated crops was only significant in the spring, when more fertilizer is applied. The amount of precipitation in the 2 weeks preceding data collection was also significant for some water quality parameters. The variation between seasons as well as the significance of precipitation to the correlations again implies that the timing of sampling campaigns may influence the correlations. Geologic parameters, such as depth to bedrock, depth to water table, slope, and soil type, were also significantly correlated to water quality parameters. Stepwise regression of independent variables and water quality indicators showed that different relationships were developed in the fall and spring. However, many of the independent variables within the stepwise regression relationships were the same for both seasons, indicating that some geologic or LULC parameters seem to consistently predict water quality. In the predictive relationships, topographic and geologic parameters occurred with the same or greater frequency as LULC parameters. Comparison of the water quality index with the biotic index demonstrated that these two indexes were best correlated during the spring, implying that the lower water quality conditions observed in the spring might be more representative of the longer-term water quality conditions in these watersheds. The correlation of turbidity, *E. coli*, and BI in the PCA analysis suggests that livestock grazing may adversely affect water quality in this watershed. PCA analysis also revealed that N, P, and SC contribute greatly to the observed water quality variability.

This study produced several practical implications: (1) sampling time, including both season and time since precipitation, may significantly impact correlations between water quality and LULC or geologic factors. Thus, timing should be a key aspect of the experimental design for field campaigns. (2) Both LULC and geologic/topographic variables are necessary to predict water quality indicators, so proposed best management practices to improve water quality should be undertaken with strong consideration of the geologic and topographic conditions of each site. Promoting best management practices in those watersheds that are most likely to be impaired (based upon geologic or topographic parameters) could help maximize the environmental benefit, with the least outlay of financial resources. (3) Although stepwise regression equations between water quality indicators and independent variables changed with the season, some independent variables were valuable predictors of water quality regardless of the season. This suggests that it may be possible to partially predict water quality indicators based on other factors, such as topographic, geologic, and LULC information. Predictive relationships cannot be used to provide specific values for water quality parameters but may be helpful for targeting sampling campaigns in streams most likely to experience impairment. This could create more efficient regulatory monitoring and improve resource allocation for water management. (4) The biotic index correlated most with parameters often associated with agriculture or urban runoff (i.e., N, P, turbidity) and was only weakly correlated with the WQI, calculated using Cude’s (2001) generally accepted method. This implies that macroinvertebrate assessment could help to distinguish LULC inputs independently from physicochemical water parameters, and that other methods of calculating the WQI might be needed to better predict biological responses based on physicochemical properties.
